# Dietary Exposure to Acrylamide and Associated Health Risks for the Korean Population

**DOI:** 10.3390/ijerph17207619

**Published:** 2020-10-19

**Authors:** Sanghee Lee, Hyun Jung Kim

**Affiliations:** 1 Research Group of Natural Materials and Metabolism, Korea Food Research Institute, Wanju-gun 55365, Korea; shlee@kfri.re.kr; 2 Research Group of Consumer Safety, Korea Food Research Institute, Wanju-gun 55365, Korea; 3 Department of Food Biotechnology, University of Science and Technology, Daejeon 34113, Korea

**Keywords:** acrylamide, food products, dietary exposure, risk assessment, margin of exposure

## Abstract

The aim of the study was to estimate the dietary exposure to acrylamide (AA) from the consumption of various processed food and to assess the associated health risks in different age groups in Korea. Potato crisps and French fries presented the highest mean levels of AA (546 and 372 μg/kg, respectively) followed by coffee (353 μg/kg) and tea products (245 μg/kg). The mean AA dietary exposure values for toddlers (≤2 years), children (3–6 years), children (7–12 years), adolescents (13–19 years), adults (20–64 years), and seniors (≥65 years) were estimated to be 0.15, 0.13, 0.06, 0.06, 0.08, and 0.06 μg/kg body weight (BW)/day, respectively. Based on the benchmark dose lower confidence limit (BMDL_10_) of 0.18 and 0.31 mg/kg BW/day, the calculated mean and 95th percentile values for the margin of exposure were below 10,000 for the all age groups suggesting possible health concern for Koreans. Biscuits, crisps, and coffee were the primary foods contributing to dietary AA exposure among these in the Korean populations. In children, considering the health risk of AA dietary exposure, especially from biscuits and crisps, there is a need to further control and modify dietary habits to ensure lower AA exposure.

## 1. Introduction

Acrylamide (AA) is an industrial chemical used for the manufacturing of polyacrylamides and has also been detected in a wide range of food products from very low levels of microgram per kilogram up to levels of a few milligram per kilogram [1,2]. The presence of variable AA amounts in food was first highlighted in 2002 [3]. When starchy foods, such as potatoes and cereal products are cooked or processed at temperatures above 120 °C by frying, roasting, or baking, heat-induced reactions between the amino group of the free amino acid asparagine and the carbonyl groups of the reducing sugars such as glucose in the food results in the formation of AA [4,5,6].

The major sources of AA in the human diet are potato- and cereal-based products as well as roasted coffee. According to a European Food Safety Authority (EFSA) statement, the highest levels of AA were observed in solid coffee substitutes (dry) and dry coffee, with average medium bound (MB) AA levels of 1499 and 522 μg/kg, respectively, followed by potato fried products. The average medium bound (MB) level of AA in a potato crisps and snacks was reported to be 389 μg/kg [7]. According to the report of Commission Regulation (EU) 2017/2158, which established mitigation measures and benchmark levels for the reduction in the presence of AA in food products [8].

Several studies have focused on investigating the toxicology of AA, and assessing the health risks of dietary exposure to AA in different food products [9,10,11,12]. The Food and Agriculture Organization/World Health Organization (FAO/WHO) reported that dietary AA exposure can vary between 0.3 and 0.8 μg/kg BW/day [9]. The dietary exposure levels for adults estimated to be 0.08 μg/kg BW/day for Korean, 0.22 μg/kg BW/day for Japanese, 0.29 μg/kg BW/day for Chinese, and 0.31 to 1.1 μg/kg BW/day in Europe [13,14,15,16].

In a Netherlands cohort study on diet and cancer, increased risks of postmenopausal endometrial and ovarian cancer were reported with an increase in dietary AA intake [11]. Another report suggested that prediagnostic exposure to acrylamide, measured by AA-hemoglobin and glycidamide-hemoglobin adducts, could be related to mortality among breast cancer patient, especially those with endocrine-related types of breast cancer [12]. Furthermore, in 2015, the European Food Safety Authority (EFSA) adopted an opinion on AA in food, confirming that AA in food potentially increases the risk of developing cancer among consumers in all age groups, whereas based on current levels of dietary exposure, the possible harmful effects of AA on the nervous system are negligible.

Since AA is present in a wide range of everyday foods, the related concerns apply to all consumers, especially to children, who are the most heavily exposed age group on a body-weight basis [7]. In light of these concerns, the reliable risk characterization of dietary exposure to AA has grown in importance in recent years. The Joint FAO/WHO Expert Committee on Food Additives [17] proposed two different BMDL_10_ (lower limits on the benchmark dose for a 10% response) for AA: 0.31 mg/kg BW/day for the induction of mammary tumors in rats and 0.18 mg/kg BW/day for Harderian gland tumors in mice. In its recent evaluation, the JECFA applied a margin of exposure (MOE) to its health risk assessment of AA to consider the possible safety concerns arising from the presence of substances that are both genotoxic and carcinogenic in food [17,18]. In Korea, monitoring and dietary exposure based on nonparametric probabilistic model of AA have been steadily reported [13]. The MOE is the most promising model due to its capability in identifying in certainties and variabilities in the long-term exposure estimations as well as in performing risk predictions that are used extensively in food [19]. However, information on dietary exposure to AA and health risk assessment with respect to the estimation of the MOE for Korean populations is limited.

Therefore, the aim of this study was to assess the dietary exposure of the Korean population to AA and identify the food products that are the main sources of exposure to AA, especially from a preventive public health perspective. Finally, the health risk of dietary exposure to AA were estimated using MOE approaches based on the BMDL_10_ of AA for the induction of mammary tumors in rats and Harderian land tumors in mice. Further, the dietary exposure distribution and related potential risk for AA ingestion were estimated by a Monte Carlo simulation.

## 2. Materials and Methods

### 2.1. Collection of Samples

Based on our previous literature [20], sample was selected according to the high levels of AA contamination. In total, this study used 485 samples of food products including potato crisps (*n* = 40), crisps (except potato crisps, *n* = 30), biscuits (*n* = 70), French fries (*n* = 40), chocolate products (*n* = 20), cocoa products (*n* = 20), breakfast cereals (*n* = 40), tea products (*n* = 25), nut and nut products (*n* = 20), dried and roasted seaweed (*n* = 20), coffee substitutes (*n* = 60), bread (*n* = 20), cakes (*n* = 40), juices (*n* = 20), and other products (kimchi, *n* = 20). Samples were collected from randomly selected local supermarkets, stores, and fast-food restaurants all over Korea in 2011.

### 2.2. Sample Preparation and Analysis

Samples were extracted and analyzed based on the FDA method for the analysis of AA in food [21]. Briefly, a 1 ± 0.1 g sample was weighed in a 50-mL polypropylene tube, and 1 mL of 200 ng/mL ^13^C_3_-AA solution and 9 mL of water were added. After mixing the solution for 20 min on a rotating shaker, the suspension was centrifuged at 5000 rpm for 15 min. The clarified aqueous supernatant (5 mL) was placed in a Maxi-Spin filter tube (0.45 μm, PVDF) and centrifuged at 6500 rpm for 5 min. The filtrate (1.5 mL) was loaded onto an OASIS HLB solid-phase extraction (SPE) cartridge (200 mg/6 mL), conditioned with 3.5 mL of methanol, followed by 3.5 mL of water. Upon elution with water, the first 2 mL was discarded, and the ensuing portion (1.5 mL) was collected. The obtained portion was passed through a Bond Elut AccuCAT SPE cartridge (200 mg/3 mL) and conditioned with 2.5 mL of methanol, followed by 2.5 mL of water. In this step, the first 0.5 mL of the eluate was discarded, and the ensuing portion (1 mL) was collected.

The LC-MS/MS system consisted of an Agilent 1200 pump LC (Agilent Technologies, Santa Clara, CA, USA) and 4000 Q TRAP mass spectrometer (AB SCIEX, Foster City, CA, USA). Analytical separation was carried out on dC_18_ Fortis column (100 × 2.1 mm i.d., 1.7 μm; Fortis Tech., Seoul, Korea). The mobile phase employed for the isocratic elution of the analyte was a mixture of 0.5% methanol/0.1% acetic acid in water, and the flow rate was 100 μL/min. The total run time of the chromatograms was 10 min and the retention time of AA and ^13^C_3_-AA was about 6 min. The injection volume was 10 μL. The column was operated at ambient temperature. Positive ionization was performed using the following settings: ion spray voltage, 5500 V; curtain gas, 25 (arbitrary units); GS1 and GS2, 50 and 60 psi, respectively; and probe temperature, 500 °C. The multiple reaction monitoring (MRM) mode was used for ion detection and the transitions from 72 to 55 *m*/*z* for AA and from 75 to 58 *m*/*z* for the deuterated analog were monitored.

Some of our AA data (274 processed foods) for the 485 processed foods were previously published [22], whereas the rest of the data were reported by the Korea Food and Drug Administration [23]. The concentration of AA in 485 food samples, including potato crisps, crisps (other than potato crisps), biscuits, French fries, coffee, bread, cakes, breakfast cereals, chocolates products, cocoa products, nuts and nut products, juice, tea products, kimchi, and dried and roasted seaweed were analyzed.

### 2.3. Food Intake Data

To assess the Korean population’ dietary AA exposure, food intake data were obtained from the Korea National Health and Nutrition Examination Survey (KNHANES) 2008 conducted over 3 years (2007–2009) using a rolling sampling design involving a complex, stratified, multistage, and probability-cluster survey of a representative sample of the noninstitutionalized civilian population of South Korea [24]. The KNHANES is a statutory survey of people’s health behavior, the status of chronic diseases, and the actual condition of food and nutrient consumption. Food consumption data were extracted in g/person/day for the toddlers (≤2 years), children (3–6 years), children (7–12 years), adolescents (13–19 years), adults (20–64 years), and seniors (≥65 years).

### 2.4. Dietary Exposure to AA

Dietary exposure to AA was estimated for different age groups in the Korean population. AA dietary exposure (DE) was expressed as the μg/kg BW/day for each age group and estimated as follows:

$$Daily\;dietary\;exposure=\overset{n}{\underset{i=1}{\sum }}\left(\frac{Ci\times Ai}{B}\right)$$

*Daily dietary exposure*: Estimated daily dietary exposure to AA (μg/kg BW/day)
*C_i_*: the concentrations of AA in a food composite sample (μg/kg)
*A_i_*: the consumption amount of a food group for the corresponding population groups of different ages and genders, as in the KNHANES (g/day)
*B*: the body weight (kg) of the corresponding population groups obtained from KNHANES
*n*: the total number of food groups consumed.

The percentile estimation of dietary exposure to AA was based on Monte Carlo simulations using the @Risk program (Palisade, New York, NY, USA) with an iteration number of 200,000. We selected the distribution functions under the hypothesis that any number of possible AA concentrations will have an equal probability for each outcome occurring and that the values for the *C_i_*, A*_i_*, and B parameters. For the *C_i_* and A*_i_* parameters, distributions were determined using BestFit function provided by @Risk program. We used lognormal distribution for the B parameter.

### 2.5. Risk Assessment of AA

To evaluate the carcinogenic risk correlated to AA dietary exposure, the margin of exposure (MOE) approach based on experimental animal data was applied, which is a common approach for the risk characterization of substances that are both carcinogenic and genotoxic. The Joint FAO/WHO Expert Committee on Food Additive (JECFA) proposed two different BMDL_10_ (the lower limit of the benchmark dose for a 10% response) values for AA: 0.31 mg/kg BW/day for the induction of mammary tumors in rats and 0.18 mg/kg BW/day for Harderian gland tumors in mice [17]. For the risk assessment of dietary exposure to AA, the MOE values were calculated as the ratio between the toxicological reference (BMDL_10_) and dietary exposure to AA for all age groups [8,19,25]. Statistical analysis was performed using one-way ANOVA test and Tukey’s post hoc test using SPSS 20 (SPSS Inc., Chicago, IL, USA). The level of significance was set as 0.01.

## 3. Results and Discussion

### 3.1. Contents of AA in the Food Sample

The contents of AA in the 485 food samples purchased from the Korea market are illustrated in Table 1. Among the food groups, the highest mean AA content was found in potato crisps (546 µg/kg) and ready-to-eat (RTE) French fries (372 µg/kg, followed by coffee (353 µg/kg), tea products (245 µg/kg), biscuits (192 µg/kg), crisps (135 µg/kg), dried and roasted seaweed (114 µg/kg), breakfast cereals (80 µg/kg), and chocolates (58 µg/kg). For other food products, the mean AA content was in the range of less than the limit of quantification (LOQ) to 23 µg/kg. The AA contents in kimchi samples were lower than the LOQ (10 µg/kg).

Among these samples, some of the food categories showed 100% contamination with AA, potato crisps (*n* = 40), French fries (ready-to-eat, *n* = 40), and coffee samples (*n* = 60). Other types of foods, such as breakfast cereals (39/40 (the number of samples contaminated with AA/total number of analyzed samples), 98%), biscuits (58/70, 83%), crisps (23/30, 77%), chocolate products (13/20, 65%), tea (16/25, 64%), and nut and nut products (9/20, 45%) were also contaminated with AA. Interestingly, dried and roasted seaweed showed a high level of contamination with AA, 17 out of 20 samples (85%) were contaminated with AA. Bread, juices, cocoa products, and cake samples presented a 5% contamination ratio.

In total, 66% of the analyzed samples were contaminated with AA with a contamination level above the LOD, whereas concentration of AA in 165 of the analyzed samples (34%) were below the LOQ. Contamination with AA was above the LOQ in potato crisps, crisps (except for potato crisps), biscuits, bread, French fries, cereals, and coffee but only 8.2% of the samples (40 out of 485) exceeded the benchmark levels for AA [8]. On the EU regulation No. 2017/2158, a benchmark level of 500, 750, 400, 350, 400, 850, 50 to 100, and 150 to 300 μg/kg have been settled for French fries, potato crisps, crisps (exception of potato crisps), biscuits, roasted coffee, instant coffee, soft bread, and breakfast cereals, respectively. No benchmark levels have been set for tea, juice, cocoa and chocolate products, and nut products [8]. A total of 9 samples exceed the benchmark level set by the EU in the potato crisps (750 μg/kg) and French fries (500 μg/kg). Moreover, the AA contents of 10, 4, 6, and 2 samples exceeded in biscuits, crisps, coffee, breakfast cereals, respectively.

The content of AA ranged widely from LOQ to 1435 μg/kg. These results are similar or lower to some of the previous investigations from the literature [8,17,26,27,28]. The AA contents in this study was similar to that of the EFSA report, whereas the highest level of AA was detected in instant coffee (674 μg/kg), potato crisps (654 μg/kg), and French fries (367 μg/kg) [7]. These differences in AA content reflect the influence of different amounts of asparagine, glucose, and fructose in raw materials and the applied technological process, especially for potato crisps, French fries, coffee, nut products, dried and roasted seaweed, and tea products [18,29,30,31].

### 3.2. Dietary Exposure to AA

The estimate dietary exposure to AA from the consumption of processed food is included in Table 2 using the food consumption data obtained from KNHANES (2008) and the contamination of AA in foods analyzed in this study. The estimated dietary exposure to AA for individual age groups of the Korean population is presented in Table 2. The overall mean and 95th percentile values for the AA exposure of all populations were found to be 0.08 and 0.12 µg/kg BW/day, respectively. Dietary exposure was found to be higher in the younger (≤2 to 6 years) age groups compared to the other age groups (7 years and above). The average AA exposure decreased significantly with an increase in age, i.e., the mean dietary exposure to AA for the youngest age group (≤2 years old) and for the eldest age group (≥65 years old) was 0.15 and 0.06 µg/kg BW/day, respectively. The 95th percentile values of AA intake also decreased with an increase in age, from 0.37 µg/kg BW/day in the ≤2-year-old age group to 0.10 µg/kg BW/day in the ≥65-year-old age group. The EFSA [16] estimated the mean dietary exposure to AA of 0.3–1.1 µg/kg BW/day in the adult (>18 years) population, 0.4–1.4 µg/kg BW/day for adolescents (11–17 years), 0.7–2.1 µg/kg BW/day for children (3–10 years), and 1.2–2.4 µg/kg BW/day for toddlers (1–3 years). Therefore, the measured exposure values obtained in the present data are distinctly lower than that those in Western countries [16,32].

The median and highest (95th percentile) dietary exposure levels to AA from the consumption of each food group among the six different age groups of the Korean population are shown in Table 3 and Table 4. Biscuits, crisps, and juices are the primary processed food sources of AA for the youngest age group (≤2 years old). These three food categories comprise 78.0%, 71.0%, 52.8%, and 49.6% of the median dietary exposure in the ≤2-, 3–6-, 7–12-, and 13–19-year-old groups, respectively (Table 3). For the highly-exposed group represented by the 95th percentile values of dietary exposure to AA, exposure to AA from the consumption of the above three food categories totaled 83.8%, 79.6%, 67.1%, and 67.1% for the ≤2-, 3–6-, 7–12-, and 13–19-year-old groups (Table 4), respectively. These results suggest that biscuits, crisps, and juices are the primary sources of dietary exposure to AA in infants, toddlers, children, and adolescents in Korea. For the 7–12-year-old and 13–19-year-old age groups, the median values for the dietary exposure to AA from the consumption of potato crisps comprise 17.8% and 18.8% of the entire dietary exposure to AA (Table 3). For the 95th percentile values of dietary exposure to AA, potato crisps comprise 9.4% and 10.0% of the entire dietary exposure to AA for the age groups (Table 4).

For the 20–64-year-old and 65 years and above age groups, coffee consumption comprises 82.9% and 94.9% of the mean dietary exposure to AA (Table 3). Based on the 95th percentile values for the dietary exposure to AA, coffee consumption comprises 64.0% and 84.0% of the mean dietary exposure to AA for the 20–64-year-old and 65 years and above age groups (Table 4).

In the Polish population, similar to this study, coffee was the primary source of dietary exposure to AA in adult [23]. The contribution of biscuits and crisps to the dietary exposure to AA in this study were higher than contribution of potato products including potato crisps and French fries in the younger age group (less than 19 years old). Conversely, most AA exposure was contributed by potato crisps, French fries, and bread in previous studies [10,33,34].

For the AA contents in each food category, we observed the highest AA content in potato crisps followed by French fries and coffee with mean contamination levels of 546, 372, and 353 μg/kg, respectively (Table 1). We also observed the highest contamination level of AA in potato products, thus, the discrepancies between the highest contributing food categories for dietary exposure to AA determined in this study and those determined in previous studies could be primarily attributed to differences in consumption habits. Higher dietary exposure to AA was observed among the younger generations (toddlers and children) for both the median and 95th percentile values of dietary exposure, which is certainly due to the distinct dietary habits of younger consumers as well as the higher food intake per kilogram of body weight among younger generations compared to adults [16].

The contribution pattern of dietary AA exposure among the Korean population differ from those of other international studies. In a recent EFSA assessment report [35], the four major contributors of AA exposure among adults were fried potatoes (12.9–64.6%), unspecified bread (0.2–59.3%), soft bread (0.0–21.6%) and biscuit (0.0–12.1%). This difference could be explained by the different dietary habits between Korea and most Western countries. The Korean dietary pattern is dominated by vegetables and cereals, and is inconsistent with the diets of most Western countries.

### 3.3. Risk Assessment of AA

**Figure 1:**
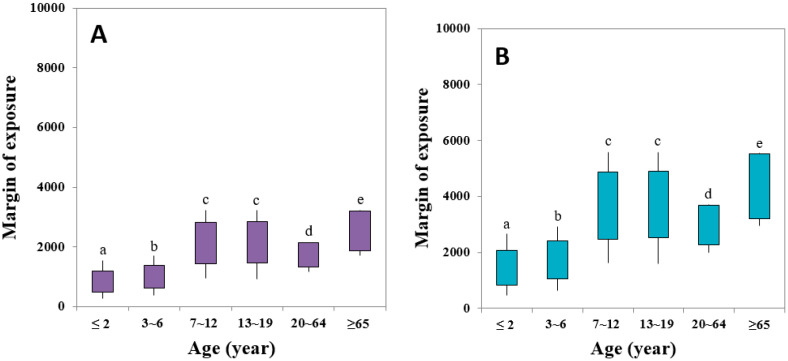
Margin of exposure (MOE) of age classes with acrylamide intake compared to benchmark dose lower confidence limit (BMDL_10_) ((**A**) Harderian gland tumors in mice and (**B**) mammary tumors in rats). Upper end of the line: 50th percentile value, lower end of the line: 99th percentile values, upper end of the box: mean value, and lower end of the line: 95th percentile values. Statistical analysis was performed using one-way ANOVA test and Tukey’s post hoc test using SPSS 20 (SPSS Inc., Chicago, IL, USA). Alphabet (a–e) in the Figure indicates that the 50th percentile values of MOE with a same letter are not significantly different (*p* < 0.01).

The MOE approach provides an indication of the level of health concern about a substance’s presence in food without quantifying the risk. The use of the MOE can help to keep exposure to such substances as low as possible [7]. As shown in Table 5, the MOE calculated for the overall mean values of dietary exposure to AA were 2347 and 4042 for both BMDL_10_ values (0.18 and 0.31 mg/kg BW/day), respectively. As shown in Figure 1A, the mean values of the MOE calculated from the dietary intake values for the six different age groups and the BMDL_10_ values (0.18 mg/kg BW/day) for the Harderian gland tumors were 1200, 1395, 2824, 2844, 2146, and 3210 for the age groups of ≤2, 3–6, 7–12, 13–19, 20–64, and ≥65 years old, respectively. For the 95th percentile MOEs, the values decreased to 485, 314, 142, 1470, 1328, and 1866 for the age groups of ≤2, 3–6, 7–12, 13–19, 20–64, and ≥65 years old, respectively. For the BMDL_10_ values (0.31 mg/kg BW/day) of mammary tumors, the calculated mean values of the MOE were 2066, 2403, 4864, 4898, 3696, and 5528 for the age groups of ≤2, 3–6, 7–12, 13–19, 20–64, and ≥65 years old, respectively. For the 95th percentile MOEs, the values decreased to 836, 1058, 2483, 2532, 2287, and 3213 for the age groups of ≤2, 3–6, 7–12, 13–19, 20–64, and ≥65 years old, respectively.

The MOE values obtained in the current study are much higher than those suggested by the EFSA (50–425) and JECFA (45–310). However, they remain lower than the critical MOE of 10,000 [35]. Generally, MOE values greater than 10,000 are considered to indicate a low risk of carcinogenic and genotoxic substances [7]. Therefore, since the MOEs for genotoxic and carcinogenic effects evaluated across all age groups considered here are lower than the critical MOE of 10,000, a possible health concern related to cancer-related effects can be highlighted. An identical result was previously reported for adults and children over 3 years of age in France [31]. The obtained MOE values in that study indicated that the genotoxic and carcinogenic risk from the dietary exposure to AA cannot be excluded for the Korean population especially for children and younger populations due to the insufficient value of the MOE and concluded that continuous efforts are necessary to reduce dietary AA intakes from the perspective of public health.

## 4. Conclusions

Among the 485 samples comprising 15 different food categories, the products that contained the highest level of AA were potato crisps, French fries, coffee, tea products, biscuits, crisps, dried and roasted seaweed, breakfast cereals, and chocolates products. Overall, 66% of the samples were contaminated with AA, but only 14% of the samples exceeded the indicative values provided by EU legislation. Based on the mean values of the estimated dietary exposure to AA, dietary exposure to AA in toddlers was approximately twofold higher than that of adults, due to their higher food intake per body weight. Considering the intake amount, biscuits and crisps, despite having lower amounts of AA than potato crisps and French fries, were the most important contributors among the younger groups in this study. Coffee was also an important source of AA dietary exposure in adult. The calculated mean and 95th MOE values for dietary exposure to AA are a health concern, and all age groups should limit their consumption of these products. To mitigate the health effects due to dietary AA exposure, especially in children, there is a need for further control and modify dietary habits and food processing to decrease AA exposure.

## Figures and Tables

**Table 1:** Acrylamide contents and estimated daily exposure in food item.

Food Category	Sample (*n*)	Acrylamide (μg/kg)		Benchmark Level ^(2)^ (μg/kg)	Estimated Daily Exposure (μg/kg BW/day)
		Mean ± SD ^(1)^	Range		
Potato crisps	40	546 ± 353	14–1435	750	0.002
Crisps (except potato crisps)	30	135 ± 176	<LOQ–693	400	0.004
Biscuits	70	178 ± 201	<LOQ–861	350	0.009
French fries	40	372 ± 220	93–1080	500	0.001
Chocolate products	20	58 ± 71	<LOQ–232	- ^(^^3)^	0.001
Cocoa products	20	4 ± 17	<LOQ–74	-	≈0 ^(4)^
Breakfast cereals	40	80 ± 82	<LOQ–370	150–300	0.001
Tea products	25	245 ± 314	<LOQ–889	-	≈0
Dried and roasted seaweed	20	114 ± 110	<LOQ–335	-	0.002
Nut products	20	23 ± 38	<LOQ–135	-	≈0
Coffee ^(5)^	60	353 ± 270	57–989	400–850	0.021
Bread	20	1 ± 4	<LOQ–17	50–100	≈0
Cakes	40	1 ± 5	<LOQ–27	50	≈0
Juice	20	8 ± 37	<LOQ–170	-	0.001
Kimchi	20	<LOQ	<LOQ	-	0

^(1)^ The mean was calculated by treating the values below the LOQ as zero. ^(2)^ The document reports benchmark levels for the presence of AA in foodstuff by EU regulation No. 2017/2158. ^(3)^ No benchmark levels have been set on the EU regulation No. 2017/2158. ^(4)^ Less than 0.001 μg/kg BW/day. ^(5)^ Coffee include roasted coffee (*n* = 40) and instant coffee (*n* = 20). The mean ± SD (range) of both coffee samples are 195 ± 99 (57–398) and 669 ± 218 (57–398) μg/kg for roasted coffee and instant coffee, respectively.

**Table 2:** Estimated dietary exposure to acrylamide among subpopulations of different ages.

Subpopulation of Different Ages (years)	Dietary Exposure to Acrylamide (mg/kg BW/day)				
	Mean	50th Percentile	90th Percentile	95th ^(1)^ Percentile	Maximum
≤2	0.150	0.116	0.273	0.371	0.659
3–6	0.129	0.106	0.226	0.293	0.493
7–12	0.064	0.056	0.102	0.125	0.192
13–19	0.063	0.056	0.099	0.122	0.194
20–64	0.084	0.084	0.128	0.136	0.156
≥65	0.056	0.056	0.091	0.096	0.105
Total	0.077	0.076	0.112	0.122	0.153

^(1)^ 95th percentile (high exposure).

**Table 3:** Dietary exposure to acrylamide among the subpopulations of Korean based on the median values of dietary exposure.

Food Category	Median Values of Dietary Exposure to Acrylamide (10^−3^ μg/kg BW/day) among the Subpopulations of Korea ^(1)^					
	Age (≤2)	Age (3–6)	Age (7–12)	Age (13–19)	Age (20–64)	Age (≥65)
Potato crisps	2.9 ^(2)^	3.8	7.6	7.9	1.1	0.0
Crisps (except potato crisps)	18.3	19.0	10.0	6.7	1.3	0.2
Biscuits	34.0	24.0	8.1	8.5	3.1	0.6
French fries	1.5	0.0	2.2	2.6	0.9	0.0
Coffee	0.0	0.4	0.0	2.1	63.3	50.8
Bread	0.5	0.7	0.6	0.4	0.2	0.0
Cakes	4.1	4.8	2.7	2.5	1.1	0.3
Cocoa products	0.0	0.0	0.0	0.0	0.0	0.0
Chocolates	3.9	4.5	3.7	3.3	0.8	0.4
Cereals	2.6	4.1	2.0	1.4	0.4	0.0
Teas	0.0	0.0	0.0	0.0	0.4	0.1
Kimchi	0.0	0.0	0.0	0.0	0.0	0.0
Juices	14.0	9.7	4.5	5.7	2.9	0.4
Dried and roasted seaweed	2.9	2.8	1.3	0.8	0.7	0.5
Nuts and nut products	0.3	0.5	0.2	0.3	0.3	0.1
Total	85.0	74.2	42.6	42.3	76.4	53.5

^(1)^ Median values of dietary exposure for each food were calculated for the consumers of each food only. However, the total corresponds to all respondents. ^(2)^ In exposure estimations, samples with acrylamide concentrations lower than the limit of detection (LOD) were assumed to contain half the LOD of acrylamide and samples with acrylamide concentrations between the LOD and the LOQ were assumed to contain half the LOQ, whereas samples likely to contain no acrylamide at all (like nonheated products) were assumed to have an acrylamide concentration of 0.

**Table 4:** Dietary exposure to acrylamide among the subpopulations of Korean based on the 95th percentile values of dietary exposure.

Food Category	95th Percentile Values of Dietary Exposure to Acrylamide (10^−3^ μg/kg BW/day) among the Subpopulations of Korea ^(1)^					
	Age (≤2)	Age (3–6)	Age (7–12)	Age (13–19)	Age (20–64)	Age (≥65)
Potato crisps	11.0 ^(2)^	13.2	18.0	18.9	2.7	0.1
Crisps (except potato crisps)	84.8	86.7	45.1	30.5	5.7	1.3
Biscuits	281.5	199.2	66.3	71.1	25.3	5.2
French fries	5.8	0.0	7.0	8.9	2.5	0.0
Coffee	0.0	2.0	0.0	4.4	112.4	91.0
Bread	1.2	1.7	1.4	1.0	0.5	0.1
Cakes	7.4	8.2	4.5	4.5	1.8	0.6
Cocoa products	0.2	1.3	0.4	0.1	0.0	0.0
Chocolates	10.2	10.4	8.0	7.4	1.7	1.0
Cereals	12.3	18.2	8.7	6.3	1.6	0.1
Teas	0.0	0.4	0.0	0.6	1.7	0.7
Kimchi	0.0	0.0	0.0	0.0	0.0	0.0
Juices	60.4	61.1	17.7	26.1	10.5	2.3
Dried and roasted seaweed	33.6	32.2	14.5	9.4	8.3	5.5
Nuts and nut products	0.8	1.3	0.7	0.9	0.8	0.4
Total	509.3	436.1	192.4	190.3	175.5	108.4

^(1)^ 95th percentiles values for each food were calculated for the consumers of each food only. However, the total corresponds to all respondents. ^(2)^ In exposure estimations, samples with acrylamide concentrations lower than the limit of detection (LOD) were assumed to contain half the LOD of acrylamide and samples with acrylamide concentrations between the LOD and the LOQ were assumed to contain half the LOQ, while samples likely to contain no acrylamide at all (like non-heated products) were assumed to have an acrylamide concentration of 0.

**Table 5:** Margin of exposure (MOE) for genotoxicity and carcinogenicity due to dietary exposure to acrylamide.

Percentile	Margin of Exposure (MOE)	
	Harderian Gland Tumors	Mammary Tumors
Mean	2347	4042
50th	2371	4084
90th	1602	2759
95th	1471	2533
99th	1177	2027

MOE values are reported for the BMDL_10_ values of Harderian gland tumors (0.18 mg/kg BW/day) and mammary tumors (0.31 mg/kg BW/day).
